# Primary bone marrow lymphoma: A hematological emergency in adults with fever of unknown origin

**DOI:** 10.1002/cam4.1669

**Published:** 2018-07-09

**Authors:** Hao‐Yuan Wang, Ching‐Fen Yang, Tzeon‐Jye Chiou, Jyh‐Pyng Gau, Po‐Min Chen, Chang‐Youh Tsai, Hui‐Chi Hsu, Fu‐der Wang, Jin‐Hwang Liu, Liang‐Tsai Hsiao

**Affiliations:** ^1^ Division of Hematology and Oncology Department of Medicine Taipei Veterans General Hospital Taipei Taiwan; ^2^ Faculty of Medicine School of Medicine National Yang‐Ming University Taipei Taiwan; ^3^ Department of Pathology and Laboratory Medicine Taipei Veterans General Hospital Taipei Taiwan; ^4^ Division of Transfusion Medicine Department of Medicine Taipei Veterans General Hospital Taipei Taiwan; ^5^ Division of Allergy, Immunology, and Rheumatology Department of Medicine Taipei Veterans General Hospital Taipei Taiwan; ^6^ Division of General Medicine Department of Medicine Taipei Veterans General Hospital Taipei Taiwan; ^7^ Division of Infectious Disease Department of Medicine Taipei Veterans General Hospital Taipei Taiwan

**Keywords:** fever of unknown origin, hemophagocytic lymphohistiocytosis, non‐Hodgkin lymphoma, primary bone marrow lymphoma

## Abstract

Primary bone marrow lymphoma (PBML) represents non‐Hodgkin lymphoma (NHL) that primarily arises in the bone marrow (BM) without lymphadenopathy. This condition has various definitions and can be masked by prolonged fever, leading to delayed diagnosis. We aimed to identify clinical features and risk indicators of PBML. We enrolled 269 adults with fever of unknown origin (FUO) who underwent a BM study for potential PBML. Thirty patients were diagnosed with PBML (26 and 4 patients in the training and validation cohort, respectively), and 20 patients (67%) showed initial manifestation of hemophagocytic lymphohistiocytosis (HLH). Among PBML patients in the training cohort, their median overall survival is short (8 days), with pneumonia being the most common direct cause of early mortality, followed by life‐threatening HLH. Despite extremely poor prognoses, some B‐cell PBML patients who survived 30 days after BM studies achieved long‐term survival with rituximab‐based treatment. To assist general practitioners in early PBML diagnosis when approaching adults with naïve FUO, we identified several risk indicators, including elevated serum alkaline‐phosphate levels, lowered serum immunoglobulin‐G levels, cytopenia in ≥2 lineages, and peripheral blood leukoerythroblastosis. Our recently published scoring system, which can predict hematological BM disease in FUO adults, showed excellent ability in recognizing PBML early, with high sensitivity and specificity. We conclude that PBML is a specific “clinical” phenotype of NHL; moreover, we have identified diagnostic clues for early identification of FUO adults with underlying PBML, which should be considered a hematological emergency once suspected in any adult with FUO.

## 1 INTRODUCTION

The involvement of the bone marrow (BM) in non‐Hodgkin lymphoma (NHL) is generally considered to represent systemic dissemination from a primary nodal site. However, several recently reported cases exhibited an uncommon NHL, without lymphadenopathy but only a primary tumor in the BM—primary bone marrow lymphoma (PBML).^1^, ^2^, ^3^, ^4^, ^5^, ^6^

The current knowledge regarding PBML has mainly been derived from several clinicopathologic reports,^1^, ^2^, ^3^, ^4^, ^5^, ^6^ and most studies of PBML report two diagnostic criteria in common: pathological evidence of BM infiltration with NHL and the absence of any lymph‐node enlargement or tumor formation.^1^, ^2^, ^3^, ^4^ However, there are two major limitations to our current understanding of this condition. First, the definitions of PBML used in previous studies varied, partly due to its rarity, heterogeneous histology, and a lack of uniform pathological features.^1^, ^2^, ^3^, ^4^ Second, PBML often initially manifests as prolonged fever or fever of unknown origin (FUO), which is usually first examined by general practitioners.^1^, ^2^, ^3^, ^4^, ^5^, ^7^, ^8^, ^9^, ^10^ Hence, the diagnosis of PBML is often delayed, leading to a poor prognosis and a median overall survival (OS) of <18 months.^1^, ^2^, ^3^, ^4^ Therefore, it is essential that hematologists can recognize patients with underlying PBML at an early stage, when they are consulted regarding FUO patients.

Accordingly, in this study, we enrolled 269 adults with FUO who underwent a BM study (BMS)—mimicking patients at risk for PBML—to analyze the clinical characteristics and outcomes associated with PBML and to identify the early parameters indicating the risk of underlying PBML. In addition, we recently developed a scoring system—Bone Marrow Score—to assist nonhematologists in early identification of hematological BM diseases before BMS is performed for adults with FUO.^11^ Here we examine the use of the BM Score in early identification of PBML.

## 2 METHODS

### 2.1 Study population

Between 1 January 2006, and 31 December 2017, we consecutively included adults with naïve FUO from the Departments of Medicine and Family Medicine at Taipei Veterans General Hospital, who underwent a BMS as part of the FUO evaluation, to identify patients with PBML. Patients enrolled in the first 10 years and the last 2 years were categorized as the training and validation cohorts, respectively. The diagnosis of FUO was based on an illness duration of >3 weeks prior to diagnosis; repeated, documented body temperature of >38.3°C; and a hospital stay of 3 days without a clear diagnosis.^12^ Furthermore, to identify immunocompetent adults with naïve FUO, we excluded the following patients: those who were aged <18 years, had a known human immunodeficiency virus infection, had been recipients of solid‐organ transplants, had received active immunosuppressive therapy, had an active autoimmune disease, had a preexisting malignant hematological disease, had a preexisting malignant solid tumor, were neutropenic (white blood cell [WBC] count <1000/μL or absolute neutrophil count [ANC] <500/μL), or were hypogammaglobulinemic (immunoglobulin G < 50% of the lower normal limit).^11^, ^13^, ^14^ This study was approved by the Institutional Review Board of Taipei Veterans General Hospital, and the need for informed consent was waived.

### 2.2 Diagnostic criteria of PBML

The following criteria were used for diagnosing lymphoid neoplasia in the BM as PBML: pathologically confirmed BM infiltration with NHL, regardless of peripheral blood involvement; no evidence of lymph‐node involvement, defined as enlargement of any lymph‐node to >1 cm on imaging studies (including thoracic, abdominal, and pelvic computerized tomography [CT] scans); the absence of tumor formation; and exclusion of leukemia/lymphoma cases with primarily BM involvement, including chronic lymphocytic leukemia/small lymphocytic lymphoma, prolymphocytic leukemia, lymphoplasmacytic lymphoma, mantle cell lymphoma, splenic marginal zone lymphoma, hairy‐cell leukemia, Burkitt lymphoma, and acute lymphoblastic leukemia.^2^, ^3^, ^4^

### 2.3 Definition of HLH and leukoerythroblastosis

During hospitalization, hemophagocytic lymphohistiocytosis (HLH) was diagnosed based on the HLH‐2004 criteria.^15^ Leukoerythroblastosis in a peripheral blood (PB) smear was defined as the presence of nucleated erythrocytes and immature myeloid cells.^16^, ^17^, ^18^

### 2.4 Calculation of bone marrow score

Bone marrow scores were calculated using seven parameters, with 1.0 point for ANC <2000/μL, 1.5 points each for Hb level <10 g/dL and LDH level greater than the Upper Normal Limit (UNL), 2.0 points for platelet (PLT) count <100 × 10^3^/μL, 4.0 points for splenomegaly, 5.5 points for ferritin level >1000 ng/mL, and six points for leukoerythroblastosis in a PB smear.^11^ All the parameters should be obtained within 3 days after admission.

### 2.5 Data collection

To identify the risk indicators for underlying PBML, we recorded the clinical and laboratory parameters that were routinely assessed upon admission and/or within 3 days after admission, and selected the measurements recorded closest to admission day. For patients who underwent >1 BMS, the results were recorded as follows: if all BMS results indicated nondiagnostic outcomes, the findings of the first BMS were considered; if at least one BMS indicated a positive diagnosis, the findings of the earliest BMS with positive diagnostic findings were included.

Furthermore, for patients with early mortality, defined as an overall survival (OS) duration of <30 days, direct and contributing causes were distinguished after reviewing patient medical records. Respiratory failure was defined as the need for invasive mechanical ventilation, and renal failure was defined as the need for renal replacement therapy or the presence of Stage 3 KDIGO acute kidney injury.^19^ Hepatocellular injury was defined as the presence of a primary acute liver process or severe liver damage considered to be significant by the health‐care providers, wherein alanine aminotransferase and aspartate aminotransferase levels were >fivefold over the UNL.

### 2.6 Statistical analysis

To compare the patients with and without the parameters specified (with vs without PBML), we used *t*‐tests or Mann‐Whitney *U* tests for quantitative data, χ^2^ or Fisher exact tests for categorical data, and the Kaplan‐Meier estimate and log‐rank test for OS. OS duration was defined as the interval between the date of BMS and the date of death (from any cause) or the date of the final follow‐up (31 July 2017). Cox and logistic regression models were used to calculate hazard ratios (HRs) and odds ratios (ORs), respectively, and all candidate factors with a *P‐*value of <0.2 in univariate analyses were subsequently entered into a multivariate regression model. The HRs and ORs of each factor are reported with the corresponding *P‐*values and 95% confidence intervals (CIs). All statistical analyses were performed using SPSS, version 17.0 (SPSS Inc., Chicago, IL, USA); a two‐tailed *P‐*value of <0.05 was considered statistically significant.

## 3 RESULTS

### 3.1 Clinical features of 30 adults with PBML

**Figure 1:**
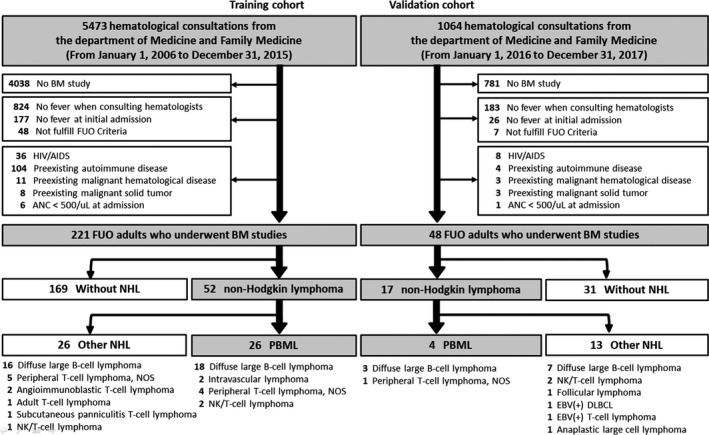
Enrollment Algorithm For Immunocompetent Adults with Naïve Fever of Unknown Origin Who Underwent a Bone Marrow Study. AIDS, acquired immunodeficiency syndrome; ANC, absolute neutrophil count; BM, bone marrow; FUO, fever of unknown origin; HIV, human immunodeficiency virus; NHL, non‐Hodgkin lymphoma

As shown in Figure 1, we included 269 adults with FUO who underwent a BMS (Table S1); of these, 30 patients were eventually diagnosed with PBML (Table S2). For the 30 diagnosed, the median age was 66 years, and 60% were male. Pathological heterogeneity exists in PBML, among which diffuse large B‐cell lymphoma (DLBCL) was the most common in the patients studied. At diagnosis, cytopenia in at least two lineages, and elevated serum lactic dehydrogenase (LDH) and ferritin levels were noted in almost every patient with PBML, followed by splenomegaly in 90%, and leukoerythroblastosis in 50% of patients. Except for two patients, all PBML patients required critical care. Of note, HLH was an initial presentation in 67% (20/30) of the PBML cases.

### 3.2 Extremely high risk of early mortality in PBML

**Figure 2:**
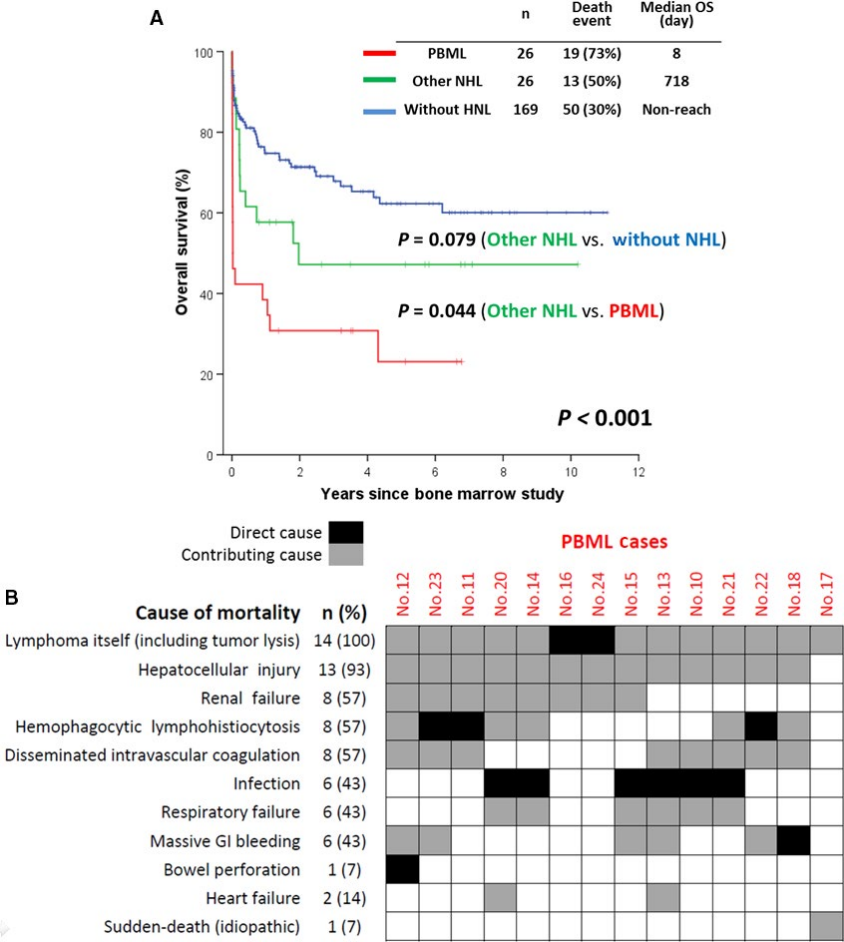
A, Overall Survival Among 221 Adults with Fever of Unknown Origin in the Training Cohort. B, Distribution of the cause of 30‐day early mortality in patients with primary bone marrow lymphoma (n = 14); the numbering of the cases represents the patients described in Table S2. NHL, non‐Hodgkin lymphoma; OS, overall survival; PBML, primary bone marrow lymphoma

Compared to the NHL patients with lymphadenopathy, those with PBML had a much poorer prognosis in the training cohort (median OS 718 vs 8 days, respectively *P *<* *0.001; Figure 2A). This suggests that more than half of the cases (14/26, 54%) had early mortality, within 30 days of the BMS. In fact, the median time needed for PBML diagnosis was 13 days after admission; moreover, five patients died before the pathological diagnosis was confirmed, and two died on the day of pathological diagnosis.

With regard to the causes of early mortality in the 14 patients (Figure 2B), pneumonia directly caused death in six cases (43%), and uncontrollable HLH led to death in three cases (21%). Two patients each experienced catastrophic tumor lysis syndrome and BM failure. One patient died of ischemia and perforation of the bowel caused by massive intestinal bleeding related to HLH and disseminated intravascular coagulation. Of note, hepatocellular injury contributed to mortality in almost all cases.

### 3.3 Role of Rituximab in treating B‐cell PBML

**Figure 3:**
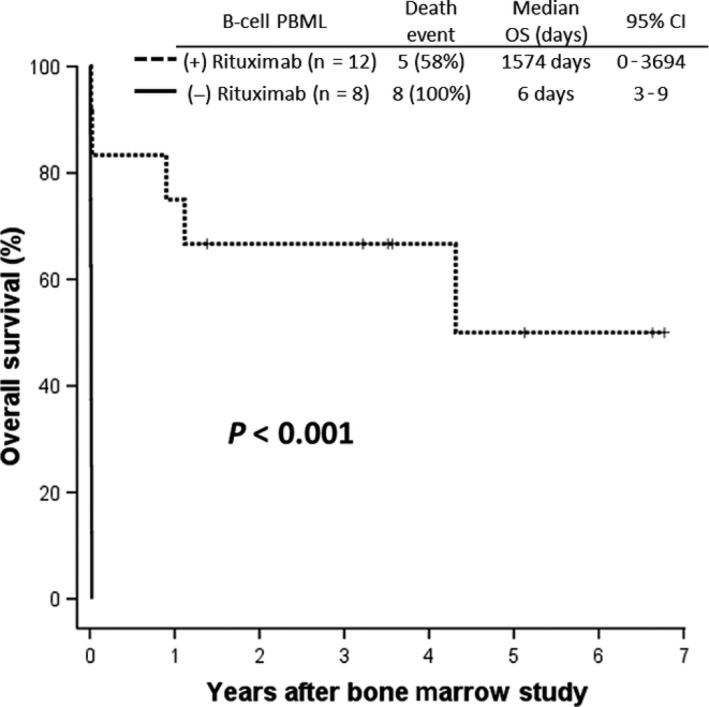
Overall Survival Among 20 Patients with B‐cell Primary Bone Marrow Lymphoma in the Training Cohort (with vs without Rituximab Treatment). CI, confidence interval; FUO, fever of unknown origin; HLH, hemophagocytic lymphohistiocytosis

Despite dismal prognoses, some B‐cell PBML patients (5 DLBCL and 1 Asian‐variant intravascular lymphoma) achieved long‐term survival, if they survived the initial high‐mortality period and received rituximab‐based treatment (Figure 3). Moreover, none of these long‐term survivors had received frontline autologous peripheral blood stem cell transplantation.

### 3.4 Risk indicators for PBML among adults with FUO

Considering the delayed diagnosis of PBML and the lack of physician awareness, we aimed to identify risk indicators of PBML. When we compared the admission parameters (Table S3), in the training cohort between FUO adults not diagnosed with PBML with those who finally were diagnosed with PBML, we found the following. PBML patients were significantly older; had lowered WBC counts, ANCs, hemoglobin levels, platelet (PLT) counts, and IgG levels; higher serum levels of LDH, ferritin, total bilirubin (T‐Bil), AST, alkaline phosphatase (ALP), and triglycerides; longer prothrombin times (PTs); and a higher incidence of splenomegaly and leukoerythroblastosis in PB smears. Moreover, trends toward higher serum gamma‐glutamyl transpeptidase (γGT) levels and higher incidence of hepatomegaly were observed.

Table 1 shows that factors with a *P*‐value of <0.2 in univariate analyses were entered into the multivariate regression model. Finally, we determined that the presence of bicytopenia or pancytopenia, leukoerythroblastosis in PB smears, IgG levels <0.67 times the UNL, and ALP levels >2 times the UNL were independent risk indicators of PBML among FUO adults, whereas PT levels >1 time the UNL and LDH levels >2 times the UNL exhibited borderline significance.

**Table 1:** Univariate and multivariate analyses of the risk indicators for primary bone marrow lymphoma in 221 adults with fever of unknown origin (Training cohort)

	Univariate		Multivariate^a^^,^ b ^,^ c	
	OR (95% CI)	*P*	OR (95% CI)	*P* g
Risk indicators for all PBML				
Age >50 y^d^	3.149 (1.044‐9.504)	0.042		
Male sex	1.490 (0.633‐3.508)	0.361		
Bicytopenia or pancytopenia^e^	22.844 (6.572‐79.404)	<0.001	20.559 (3.101‐136.279)	**0.002**
Leukoerythroblastosis on PB smear	1.390 (1.193‐1.619)	<0.001	1.384 (1.008‐1.901)	**0.045**
LDH >500 IU/L (two times the UNL)^d^	15.693 (4.543‐54.212)	<0.001	4.956 (0.832‐29.511)	0.079
Ferritin >1500 ng/mL^d^	2.216 (0.940‐5.228)	0.069		
IgG <1000 (0.67 times the UNL)^d^	4.912 (2.015‐11.972)	<0.001	5.086 (1.046‐24.728)	**0.044**
T‐Bil >1.6 mg/dL (one time the UNL)^d^	3.400 (1.372‐8.427)	0.008		
AST >45 U/L (one time the UNL)^d^	20.047 (2.662‐150.978)	0.004		
ALP >200 U/L (two times the UNL)^d^	6.000 (2.473‐14.556)	<0.001	5.857 (1.179‐29.085)	**0.031**
rGT >60 U/L (one time the UNL)^d^	2.788 (1.108‐7.015)	0.029		
PT >12 s (one time the UNL)^d^	6.416 (2.551‐16.133)	<0.001	4.457 (0.939‐21.161)	0.060
Triglyceride >133 mg/dL (0.67 times the UNL)^d^	6.338 (2.073‐19.380)	0.001		
Splenomegaly	16.063 (4.649‐55.509)	<0.001		
Hepatomegaly	2.545 (0.763‐8.492)	0.129		
Risk indicators for B‐cell PBML				
Age >50 y^d^	5.153 (1.162‐22.859)	0.031		
Male sex	1.183 (0.463‐3.024)	0.725		
Bicytopenia or pancytopenia^e^	16.884 (4.745‐60.082)	<0.001	17.870 (3.509‐91.009)	**0.001**
Leukoerythroblastosis on PB smear	1.464 (1.239‐1.730)	<0.001	8.738 (2.252‐33.905)	**0.002**
IgG <1000 (0.67 times the UNL)^d^	4.156 (1.527‐11.312)	0.005	4.688 (1.273‐17.263)	**0.020**
ALP >200 U/L (two times the UNL)^d^	3.483 (1.341‐9.049)	0.010		
Risk indicators for T‐cell PBML				
Age >50 y^d^	1.145 (0.205‐6.410)	0.877		
Male sex	3.945 (0.452‐34.398)	0.214		
Bicytopenia or pancytopenia^e^	Not converge^f^		‐	‐
Leukoerythroblastosis on PB smear	1.119 (0.776‐1.615)	0.547		
IgG <1000 (0.67 times the UNL)^d^	8.312 (1.458‐47.392)	0.017	7.059 (1.169‐42.632)	**0.033**
ALP >200 U/L (two times the UNL)^d^	8.514 (1.499‐48.356)	0.016	8.762 (1.454‐52.790)	**0.018**

Not entered into the multivariate regression model; ALP, alkaline phosphatase; ALT, alanine aminotransferase; ANC, absolute neutrophil count; AST, aspartate aminotransferase; BM, bone marrow; CI, confidence interval; CRP, C‐reactive protein; γGT, gamma‐glutamyl transpeptidase; Hb, hemoglobin; HLH, hemophagocytic lymphohistiocytosis; LDH, lactate dehydrogenase; OR, odds ratio; PLT, platelet; T‐Bil, total bilirubin; UNL, upper limit of normal; WBC, white blood cell.

a Determined using the backward method with the multivariate logistic regression model due to the small number of cases.

b Multivariate logistic regression model included all the available variables with a *P*‐value of <0.200.

c Age and sex were included into the multivariate analysis because they may confound the between‐subject comparisons.

d Receiver operating characteristics curve was used to determine the best cutoff value with maximum sensitivity and specificity.

e Cutoff value of cytopenia: WBC <4000/μL, Hb <10 g/dL, PLT <100 × 10^3^/μL

f All six T‐cell PBML had bicytopenia or pancytopenia.

g *P* value < 0.05 were presented in bold.

In the validation cohort (Table S4), PBML patients also had more severe neutropenia and thrombocytopenia, higher serum ALP level, lower serum IgG level, and more frequent leukoerythroblastosis in PB smears. All four PBML patients had IgG levels <0.67 times the UNL, ALP levels >2 times the UNL, and cytopenia in at least two lineages. Leukoerythroblastosis was noticed in three PBML patients (75%).

### 3.5 Prediction of PBML in adults with FUO using the BM score

**Figure 4:**
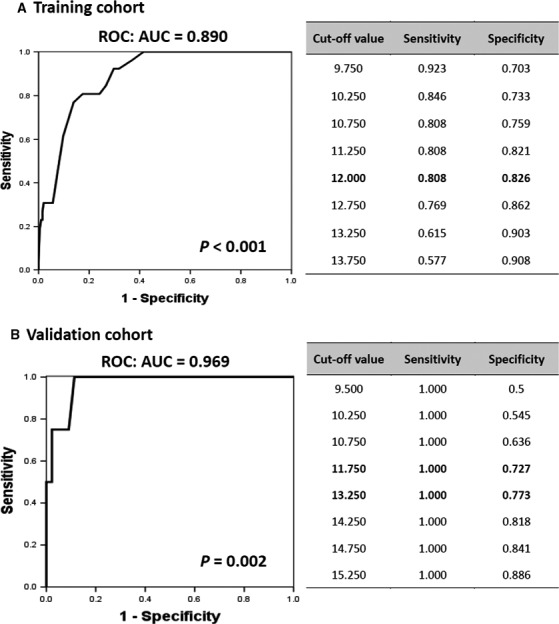
Receiver Operating Characteristic (ROC) Curve and Area Under the Curve (AUC) for Assessing the Discriminatory Power of the Bone Marrow Score for the Early Identification of Primary Bone Marrow Lymphoma in Adults with Fever of Unknown Origin in the (A) Training and (B) Validation Cohort. CI, confidence interval

Given the high occurrence of cytopenia, splenomegaly, elevated serum ferritin and LDH levels, and leukoerythroblastosis in PBML (Table S2) and the clinical utility of BM Scores in predicting hematological BM disease in FUO adults,^11^ we considered that our BM scoring system could be useful in determining PBML risk in adults with FUO. Among the 26 PBML cases of the training cohort, the median score was 14 points (Table S2). Moreover, the area under the receiver operating characteristics curve (AUC) indicated that the system had excellent discrimination ability (AUC, 0.890, 95% CI: 0.839‐0.941, *P *<* *0.001; Figure 4); with a cutoff of 12 points, its sensitivity and specificity were found to be 81% and 83%, respectively. In the validation cohort, all PBML cases had BM score more than 14 points, and AUC as high as 0.969 confirmed the discriminatory power of BM score.

## 4 DISCUSSION

To our knowledge, this is the largest case series study of PBML patients. Here, we investigated the clinical features and risk indicators of PBML considering general practitioners, who are the first to examine adults with naïve FUO, and obtained several important findings. First, we observed a close association between PBML and HLH among FUO adults, as two‐thirds (62%) of PBML patients in our FUO cohort initially exhibited HLH. Upon reviewing the literature, we summarized the case series and reports describing PBML with HLH (Table 2).^1^, ^2^, ^5^, ^8^, ^20^, ^21^, ^22^, ^23^ Similar to our findings, 60% (45/75) of the reported cases suffered from HLH, and most did not survive. Interestingly, there is a geographical prevalence of HLH‐complicated PBML in Eastern Asia, particularly Japan and Taiwan.

**Table 2:** Summary of the studies describing primary bone marrow non‐Hodgkin lymphoma with hemophagocytic lymphohistiocytosis

Author	Year	Country	No. of PBML^a^	Age	Gender (M:F)	Immunophenotype		Fever	HLH	Outcome		Ref
						B	T			Alive	Dead	
Falini et al	1990	Europe	4	53	3:1	0	4	4	4	0	4	20
Wong et al	1992	Hong Kong	11	57	7:4	4^b^	0^b^	9	6	1	10	8
Ponzoni et al	1994	U.S.A	4	68	3:1	1	3	4	1	0	4	5
Murase et al	2000	Japan	18	65	9:9	18	0	18	18	1	17	22
Gudgin et al	2005	UK	1	60	1:0	0	1	1	1	0	1	21
Kajiura et al	2007	Japan	25	66	14:11	25	0	21	7	5	20	1
Yeh et al	2010	Taiwan	11	69	8:3	11	0	11	7	1	10	2
Li et al	2014	Taiwan	1	76	0:1	1	0	1	1	1	0	23
Total			75	64	45(60%):30	60 (80%)	8 (11%)	69 (92%)	45 (60%)	9 (12%)	66 (88%)	
Wang et al (Current study)	2018	Taiwan	30	66	18(60%):12	23 (77%)	7 (23%)	30 (100%)	20 (67%)	7 (23%)	23 (77%)	

F, female; HLH, hemophagocytic lymphohistiocytosis; M, male; No., number of patients; PBML, primary bone marrow lymphoma.

a Various enrollment criteria were adopted in these studies; however, only PBML cases are included in his table.

b In Wong's study (Ref. 8), four patients had B‐cell immunophenotype, one had non‐B & non‐T immunophenotype, and the rest six patients were unavailable.

Second, our study highlighted the diagnostic dilemma presented in cases of PBML. Our diagnosed patients had a high rate (14/26 = 54%) of early mortality, and half of these (7/14) expired before the diagnosis had been made. Hence, the incidence of PBML in the historical studies may have been underestimated, because some patients may have died before BMS was performed or before they were transferred to a medical center. Given that most PBML patients were initially admitted for FUO and were cared for by nonhematologists, we emphasize that PBML should be considered a hematological emergency whenever it is suspected in any adult with FUO.

Third, we support the notion that PBML should be considered a specific “clinical” phenotype of NHL.^4^ PBML frequently manifests as HLH, with an extremely poor prognosis; however, it shows heterogeneous histology. Moreover, PBML can be identified as a special clinical phenotype in various study cohorts, including those considering cases of large cell lymphoma in the leukemic phase,^6^, ^24^ the Asian‐variant of intravascular large B‐cell lymphoma,^22^ or de novo CD5(+) DLBCL.^25^

Fourth, to identify FUO patients at risk of underlying PBML at an early stage, we determined six potential risk indicators for PBML diagnoses in adults with FUO. Among these, a lowered IgG level (a serum IgG level <two‐thirds of the UNL) might be the most informative. FUO patients with underlying infectious or inflammatory etiologies usually develop polyclonal hypergammaglobulinemia. However, a lowered IgG level in patients with PBML may suggest the presence of myelophthisis, which could lead to immunoparalysis. Regarding other risk indicators, cytopenia and elevated LDH levels were found in most studies of PBML;^1^, ^2^, ^3^, ^8^ nevertheless, we confirmed WBC <4000/μL, Hb <10 g/dL, PLT <100 × 10^3^/μL, and LDH >2 times the UNL as threshold levels. Moreover, leukoerythroblastosis in PB smears has been closely associated with hematological BM disease,^16^, ^17^, ^18^ although this easily attainable diagnostic aid is often overlooked in the current molecular era. Furthermore, consistent with the findings of several case series wherein patients with PBML had abnormal liver function test results,^5^, ^8^ we found both elevated ALP and PT levels, which may be relevant to the hepatic involvement of lymphoma and/or a concomitant complication of HLH.

Finally, we recently developed a diagnostic tool for use in adult patients with naïve FUO—Bone Marrow Score—which can predict HLH in adults with FUO.^11^, ^26^ This scoring system utilizes easily available parameters, including cell blood counts, the presence of peripheral blood leukoerythroblastosis, serum LDH and ferritin levels, and splenomegaly. Here we show the usefulness of BM Scores in early prediction of, not only HLH but also underlying PBML in FUO patients. Again, our study demonstrated the close relationship between PBML and HLH in adults with FUO.

Our study has several inherent limitations. First, the relatively small number of patients with PBML and non‐PBML NHL may have decreased the discriminatory power of some variables. Second, although it has been recommended in several reports,^10^, ^27^, ^28^, ^29^ F^18^‐fluorodeoxyglucose positron emission tomography (PET) was not routinely performed on our patients; therefore, we were unable to determine the usefulness of this tool in diagnosing PBML; however, nine of thirty PBML patients did perform the PET to confirm the absence of any lymph‐node involvement or tumor formation. Third, considering the lack of standard guidelines for treating PBML and the retrospective design of our study, it was difficult to compare different treatment modalities. However, as shown in Figure 3, we provide some evidence for a therapeutic benefit from rituximab in treating patients with PBML.

In conclusion, PBML is a specific clinical phenotype of aggressive NHL with a heterogeneous histology. It is often masked by FUO and presents as life‐threatening HLH, causing a high rate of early mortality. Given that most PBML patients are first examined by general practitioners, and that this condition is not diagnosed until after a BMS following a hematological consultation, we emphasize that PBML should be considered a hematological emergency whenever it is suspected in any FUO adult. Furthermore, our study provides diagnostic clues that could assist both general practitioners and hematologists in the early identification of FUO patients with underlying PBML.

## CONFLICT OF INTEREST

The authors declare that they have no conflict of interest.
