# Supplementary material for: Primary bone marrow lymphoma: A hematological emergency in adults with fever of unknown origin
Source: Cancer Med. 2018 Jul 9;7(8):3713–21. doi: 10.1002/cam4.1669 (PMC6089188; doi:10.1002/cam4.1669)
Supplement: Supplementary file 1 [file CAM4-7-3713-s001.docx]

| **Supplementary Table 1. Final diagnosis for 269 adults with fever of unknown origin** | |
| --- | --- |
| **Final diagnosis of training cohort** | **Patient No (%)** |
| **Neoplasm** | **83 (37.5%)** |
| Hematological malignancies | **77** |
| B-cell, non-Hodgkin lymphoma | 36 |
| T or NK-cell, non-Hodgkin lymphoma | 16 |
| Acute myeloid leukemia | 9 |
| Myelodysplastic syndrome | 5 |
| Hodgkin disease | 3 |
| Burkitt leukemia/lymphoma | 3 |
| Chronic myelomonocytic leukemia | 2 |
| Acute lymphoblastic leukemia | 1 |
| Primary myelofibrosis | 1 |
| Monoclonal gammopathy of undetermined significance | 1 |
| Solid tumors | **6** |
| Malignancy of unknown origin | 3 |
| Papillary microcarcinoma of the thyroid gland | 1 |
| Thymic carcinoma | 1 |
| Urothelial cell carcinoma | 1 |
| **Infection** | **48 (21.7%)** |
| Viral infection | 16 |
| Bacterial infection | 12 |
| Mycobacterium tuberculous | 10 |
| Non-tuberculous Mycobacterium | 5 |
| Fungal infection | 1 |
| Parasite infection | 1 |
| Common variable immunodeficiency with recurrent infection | 1 |
| Selective IgA deficiency with recurrent infection | 1 |
| Protein-losing enteropathy with recurrent infection | 1 |
| **Autoimmune, Immunology, Rheumatology** | **41 (18.6%)** |
| Adult-onset Still disease | 12 |
| Systemic lupus erythematosus | 12 |
| Hypersensitivity pneumonitis | 3 |
| Vasculitis | 2 |
| Sjogren’s syndrome with interstitial lung disease | 2 |
| Idiopathic hypereosinophilic syndrome | 2 |
| Mixed connective tissue disease | 1 |
| Churg-Strauss syndrome | 1 |
| Seronegative rheumatoid arthritis | 1 |
| Rheumatoid arthritis with CNS involvement | 1 |
| Ankylosing spondylitis | 1 |
| Gouty arthritis | 1 |
| Ulcerative colitis | 1 |
| Autoimmune hepatitis | 1 |
| **Miscellaneous** | **9 (4.1%)** |
| Drugs | 3 |
| Familial Mediterranean fever | 1 |
| Kawasaki disease, adult type | 1 |
| Kikuchi disease | 1 |
| Sweet syndrome | 1 |
| Subacute thyroiditis | 1 |
| Megaloblastic anemia | 1 |
| **No diagnosis** | **40 (18.1%)** |
| **Final diagnosis of validation cohort** | **Patient No (%)** |
| **Neoplasm** | **25 (52.0%)** |
| Hematological malignancies | **24** |
| B-cell, non-Hodgkin lymphoma | 12 |
| T or NK-cell, non-Hodgkin lymphoma | 5 |
| Hodgkin disease | 2 |
| Acute myeloid leukemia | 2 |
| Myelodysplastic syndrome | 1 |
| Primary myelofibrosis, early-stage | 1 |
| Acute lymphoblastic leukemia | 1 |
| Solid tumors | **1** |
| Prostate adenocarcinoma | 1 |
| **Infection** | **9 (18.8%)** |
| Viral infection | 3 |
| Mycobacterium tuberculous | 3 |
| Non-tuberculous Mycobacterium | 1 |
| Fungal infection | 1 |
| Leptospirosis | 1 |
| **Autoimmune, Immunology, Rheumatology** | **4 (8.3%)** |
| Adult-onset Still disease | 1 |
| Systemic lupus erythematosus | 1 |
| Anti-phospholipid syndrome | 1 |
| Takayasu disease | 1 |
| **Miscellaneous** | **2 (4.2%)** |
| Anti-interferon-gamma antibody syndrome | 1 |
| IgM monoclonal gammopathy and JAK2 V617F mutation | 1 |
| **No diagnosis** | **8 (16.7%)** |

IgA, immunoglobulin A; IgM, immunoglobulin M; NK, natural killer
