# Supplementary material for: Primary bone marrow lymphoma: A hematological emergency in adults with fever of unknown origin
Source: Cancer Med. 2018 Jul 9;7(8):3713–21. doi: 10.1002/cam4.1669 (PMC6089188; doi:10.1002/cam4.1669)
Supplement: Supplementary file 2 [file CAM4-7-3713-s002.docx]

| **Supplementary Table 2. Clinical, laboratory, and pathological features and outcomes of 30 patients with primary bone marrow lymphoma** | | | | | | | | | | | | | | | | | |
| --- | --- | --- | --- | --- | --- | --- | --- | --- | --- | --- | --- | --- | --- | --- | --- | --- | --- |
| **Training cohort** | | | | | | | | | | | | | | | | | |
| **No** | **Diagnosis** | **Age/Sex** | **BM score^a^** | **Cytopenia^b,c^** | **LEBC^b^** | **LDH^b^** | **SM/HM^b^** | **Ferritin^b^** | **HLH** | **Lymphoma cell in BM** | **Pattern^d^** | **Diagnosis Delay ^e^** | **PET ^f^** | **Treatment** | **Outcome** | **OS(D) ^g^** | **Comorbidity** |
| 1 | DLBCL | 69/M | 14 | A/T/L | Y | 2101 | Y / N | 2438 | N | 20 % | INT, DIF | 14 |  | [R-CHOP x6] | Alive/CR | 1869 |  |
| 2 | DLBCL | 49/F | 13.5 | A/T/L | N | 1840 | Y / N | 5313 | Y | 2 % | Focal clusters | 6 | Y | [R-CHOP x8] | Alive/CR | 1174 |  |
| 3 | DLBCL | 63/F | 14.5 | A/T/N | N | 2598 | Y / Y | 1090 | Y | 35 % | INT, DIF | 7 |  | [R-CHOP x8] | Alive/CR | 2471 |  |
| 4 | DLBCL | 61/M | 14.5 | A/N/L | N | 2130 | Y / N | 8967 | Y | 60 % | INT, NOD | 9 | Y | [R-CEOP x8],  [R+ HD-MTX/AraC+ IT x8] | Alive/CR | 2420 |  |
| 5 | DLBCL | 55/M | 20.5 | A/T/L | Y | 1148 | Y / N | 10112 | Y | 60 % | INT, DIF | 11 | Y | [R-CHOP + Etop + IT x2] | AWD | 503 | DM |
| 6 | DLBCL | 50/M | 13 | A/N/L | Y | 1174 | Y / N | 589 | N | 85 % | DIF | 25 | Y | [R-CEOP x8],  [R+ HD-MTX/AraC+ IT x8], [R-BEAM + Auto], [RT] | AWD | 1301 |  |
| 7 | DLBCL | 81/F | 18.5 | A/T/L | Y | 539 | Y / N | 3802 | Y | 5 % | NOD | 44 |  | [R-miniCOP] x8 | Dead^h^/CR | 328 | Alzheimer's |
| 8 | DLBCL | 81/M | 8.5 | N/T/L | N | 1625 | Y / N | 474 | N | 20 % | IS | 13 |  | [R-CHOP x6] | Dead^k^/CR | 1573 | CAD^m^; DM; HTN |
| 9 | DLBCL | 67/F | 14.5 | A/T/N | Y | 945 | Y / N | 2492 | N | 20 % | NOD | 9 |  | [R-CHOP x8] [R-ESHAP x3] [RT] | DOD | 407 |  |
| 10 | DLBCL | 83/M | 13 | A/T/N | Y | 2400 | Y / N | 1862 | N | 5 % | IS | 34 | Y | R x1 | DOD | 9 | 3°AV block^m^ |
| 11 | DLBCL | 72/M | 20.5 | A/T/N | Y | 1395 | Y / N | 2393 | Y | 15 % | NOD | 6 |  | R x1 | DOD | 5 | Afib |
| 12 | DLBCL | 57/F | 20.5 | A/T/L | Y | 1767 | Y / N | 4116 | Y | 10 % | INT, DIF | 41 |  |  | DOD | 5 | ESRD; CVA^m^ |
| 13 | DLBCL | 73/F | 10 | A/N/N | N | 848 | Y / N | 1428 | N | 15 % | IS, NOD | 8 |  |  | DOD | 8 |  |
| 14 | DLBCL | 86/M | 13 | A/T/L | N | 886 | Y / N | 1590 | Y | 50 % | NOD | 14 |  |  | DOD | 5 | COPD; HTN |
| 15 | DLBCL | 84/M | 17 | A/T/N | Y | 6168 | Y / N | 15056 | N | 15 % | INT, DIF | 33 |  |  | DOD | 7 | HTN |
| 16 | DLBCL | 50/M | 20.5 | A/T/N | Y | 2101 | Y / Y | 1873 | N | 90 % | DIF | 21 |  |  | DOD | 3 | DM; Cirrhosis^n^ |
| 17 | DLBCL | 79/F | 10.5 | A/T/N | N | 600 | N / N | 3754 | N | 20 % | IS | 10 |  |  | DOD | 2 | CVA^m^; HTN |
| 18 | DLBCL | 65/F | 12.5 | A/T/N | N | 1964 | Y / N | 3929 | Y | 30 % | INT, NOD | 13 |  |  | DOD | 1 | DM; HTN |
| 19 | AIVL | 66/M | 14 | A/T/L | N | 2854 | Y / N | 1385.4 | Y | 95 % | IV | 11 | Y | [R-CEOP + Etop x8] | Alive/CR | 1282 | Cirrhosis^n^ |
| 20 | AIVL | 71/M | 21.5 | A/T/L | Y | 1603 | Y / N | 1671 | Y | 25 % | IV | 15 |  |  | DOD | 6 | CKD; CHF; HTN |
| 21 | PTCL, NOS | 59/M | 16.5 | A/T/L | Y | 1286 | N / N | 13031 | Y | 25 % | INT | 12 |  |  | DOD | 6 |  |
| 22 | PTCL, NOS | 36/M | 14 | N/T/L | N | 2060 | Y / N | 8086 | Y | 5 % | INT | 9 |  |  | DOD | 1 |  |
| 23 | PTCL, NOS | 52/M | 13 | A/T/L | N | 704 | Y / N | 6248 | Y | 40 % | INT | 23 |  |  | DOD | 4 | CVA^m^; HTN |
| 24 | PTCL, NOS | 94/M | 10 | A/T/L | N | 1364 | N / N | 3909 | N | 75 % | INT | 13 |  |  | DOD | 8 |  |
| 25 | NK/T-cell lymphoma | 56/M | 14 | A/T/L | N | 6879 | Y / Y | 9047 | Y | 15 % | INT | 6 |  | [SMILE x2] | DOD | 34 |  |
| 26 | NK/T-cell lymphoma | 43/F | 9 | A/T/N | N | 616 | Y / Y | 1111 | Y | 70 % | INT | 16 | Y | [CHOP+Etop x4] [HD-MTX/Ara+ IT x3] [CyTBI + MU-Allo] [RT] | DOD | 381 |  |
| **Validation cohort** | | | | | | | | | | | | | | | | | |
| **No** | **Diagnosis** | **Age/Sex** | **BM score^a^** | **Cytopenia^b,c^** | **LEBC^b^** | **LDH^b^** | **SM/HM^b^** | **Ferritin^b^** | **HLH** | **Lymphoma cell in BM** | **Pattern^d^** | **Diagnosis Delay^e^** |  | **Treatment** | **Outcome** | **OS(D)^f^** | **Comorbidities** |
| 27 | DLBCL | 64/M | 21.5 | A/T/N | Y | 411 | Y / N | 33964 | Y | 25 % | NOD | 3 | Y | [R-CHOP x2] | DOD | 35 | DM |
| 28 | DLBCL | 82/F | 20 | A/T/L | Y | 608 | Y / N | 10490 | Y | 80 % | DIF | 7 |  |  | DOD | 15 | CKD; CVA^k^ |
| 29 | DLBCL | 85/F | 21.5 | A/T/L | Y | 1270 | Y / N | 4282 | Y | 5 % | NOD | 31 | Y |  | DOD | 22 |  |
| 30 | PTCL, NOS | 75/F | 15.5 | A/T/L | N | 296 | Y / N | 18813 | Y | 30 % | INT | 21 |  |  | DOD | 15 | Parkinsonism |

^a^ Calculated within 3 days after admission

^b^ At the time of diagnosing PBML

^c^ Defined the cutoff value of cytopenia: (A) anemia, Hb <10 g/dL; (T) thrombocytopenia, PLT <100 x 10^3^/µL; (L) leucopenia, WBC <4000/µL.

^d^ Infiltration pattern of lymphoma cells in BM: DIF=diffuse; INT=interstitial; IS=intrasinusoid; IV=intravascular; NOD=nodular

^e^ Defined as the interval (days) between initial admission and the confirmed diagnosis by a pathologist.

^f^ Whole-body F^18^-fluorodeoxyglucose PET scan was used to confirmed the absence of any lymph-node involvement or tumor formation after routine neck, thoracic, abdominal, and pelvic CT scans.

^g^ Defined as the interval (days) between the date of BMS and the date of death or the final follow-up.

^h^ Case No.7 died from pneumonia 4 months after completing chemotherapy.

^k^ Case No.8 died from acute myocardial infarction 4 years after completing chemotherapy.

^m^ Episodes occurred at least 3 years before the onset of PBML

^n^ Case No. 16 and 19 had liver cirrhosis relating to hepatitis C and hepatitis B, respectively, and both cases had merely Child-Pughs class A.

Afib, atrial fibrillation; AIVL, Asian variant intravascular lymphoma; Auto, autologous peripheral blood stem cell transplantation; AWD, alive with disease; BM, bone marrow; CAD, coronary artery disease; CHF, congestive heart failure; CKD, chronic kidney disease; COPD, chronic obstructive pulmonary disease; CR, complete remission; CT, computerized tomography; CVA, cerebral vascular accident; DLBCL, diffuse large B-cell lymphoma; DOD, dead of disease; DM, diabetes mellitus; ESRD, end-stage renal disease; F, female; HLH, hemophagocytic lymphohistiocytosis; HM, hepatomegaly; HTN, hypertension; IT, intrathecal triple therapy; LDH, lactate dehydrogenase; LEBC, leucoerythroblastosis; M, male; MU-Allo, matched unrelated allogenic peripheral blood stem cell transplantation; N, negative; NK, natural killer; OS, overall survival; PET, positron emission tomography; PTCL NOS, Peripheral T-cell lymphoma not otherwise specified; R, rituximab; RT, radiotherapy; SM, splenomegaly; Y, yes.
