# Supplementary material for: Primary bone marrow lymphoma: A hematological emergency in adults with fever of unknown origin
Source: Cancer Med. 2018 Jul 9;7(8):3713–21. doi: 10.1002/cam4.1669 (PMC6089188; doi:10.1002/cam4.1669)
Supplement: Supplementary file 3 [file CAM4-7-3713-s003.docx]

| **Supplementary Table 3. Early parameters of 221 adults with fever of unknown origin who were subsequently diagnosed with or without primary bone marrow lymphoma (Training cohort)** | | | | | |
| --- | --- | --- | --- | --- | --- |
| **Characteristics noted within 3 days of admission** | Immunocompetent adults with FUO^b^ | | | | *P* value^c,d^ |
|  | PBML | | | Without PBML (n=195) |  |
|  | B-cell (n=20) | T-cell (n=6) | All (n=26) |  |  |
| Lymphadenopathy, no. (%) | 0 (0) | 0 (0) | 0 (0) | 66 (34) | <0.001 |
| Age, years | 68 [58–80] | 54 [41–68] | 66 [54–79] | 58 [41–73] | 0.048 |
| Sex, male | 12 (60) | 5 (83) | 17 (65) | 109 (56) | 0.405 |
| WBC, /µL (4000–11,000^a^) | 3990 [3250–8175] | 3915 [1535–6375] | 3990 [3200–7725] | 7610 [3900–14100] | 0.005 |
| ANC, /µL (2000–5500^a^) | 3033 [1878–5865] | 1617 [1101–3782] | 2979 [1838–5595] | 4741 [2590–9901] | 0.002 |
| Hb, g/dL | 9.2 [7.3–9.9] | 10.3 [9.1–14.3] | 9.4 [8.8–10.4] | 10.5 [9.1–12.1] | 0.016 |
| PLT, × 10^3^/µL (150–400^a^) | 82 [62–141] | 55 [41–59] | 67 [53–116] | 180 [97–305] | <0.001 |
| Leukoerythroblastosis on PB smear | 10 (50) | 1 (17) | 11 (42) | 18 (9) | <0.001 |
| LDH, IU/L (131–250^a^) | 940 [570–1615] | 825 [471–1516] | 940 [558–1574] | 341 [227–634] | <0.001 |
| CRP, mg/dL (<0.5^a^) | 8.60 [5.11–13.57] | 7.72 [2.71–13.05] | 8.16 [4.76–12.68] | 6.42 [2.30–13.00] | 0.243 |
| Ferritin, ng/mL (4–274^a^) | 1784 [1064–3798] | 5079 [775–9048] | 2133 [1050–5668] | 1093 [426–3174] | 0.044 |
| IgG, mg/dL (751–1560^a^) | 1022 [851–1336] | 713 [633–1244] | 979 [768–1288] | 1380 [1090–1758] | <0.001 |
| T-Bil, mg/dL (0.2–1.6^a^) | 1.23 [0.86–2.15] | 1.05 [0.90–4.44] | 1.14 [0.89–2.29] | 0.60 [0.40–0.93] | <0.001 |
| ALT, U/L (0–40^a^) | 35 [19–58] | 87 [68–159] | 51 [21–73] | 31 [18–62] | 0.231 |
| AST, U/L (5–45^a^) | 67 [47–109] | 157 [113–286] | 84 [48–142] | 43 [22–84] | 0.001 |
| ALP, U/L (10–100^a^) | 155 [81–258] | 233 [105–372] | 186 [104–268] | 104 [70–187] | 0.003 |
| γGT, U/L (M: 8–60; F: 4–51^a^) | 101 [40–168] | 140 [86–211] | 101 [48–175] | 58 [28–117] | 0.078 |
| Na, mmol/L (135–147^a^) | 135 [130–136] | 134 [131–140] | 135 [130–137] | 136 [133–138] | 0.245 |
| PT, sec (8.0–12.0^a^) | 12.6 [12.0–13.6] | 11.8 [11.1–13.4] | 12.4 [11.7–13.4] | 11.2 [10.6–12.3] | <0.001 |
| aPTT, sec (23.9–35.5^a^) | 30.8 [27.1–35.1] | 35.5 [28.6–45.4] | 31.2 [27.6–36.5] | 31.2 [29.0–34.4] | 0.959 |
| Fibrinogen, mg/dL (200–400^a^) | 363 [277–451] | 211 [172–333] | 316 [237–425] | 336 [210–485] | 0.689 |
| Triglycerides, mg/dL (20–200^a^) | 182 [149–259] | 183 [152–218] | 182 [153–248] | 127 [88–204] | 0.008 |
| Splenomegaly | 19 (95) | 4 (67) | 23 (89) | 63 (32) | <0.001 |
| Hepatomegaly | 2 (10) | 2 (33) | 4 (15) | 13 (7) | 0.123 |

^a^ normal range

^b^ Values are reported as median [interquartile-range] or n (%)

^c^ Comparison between all PBML (n=26) and without PBML (n=195)

^d^ Determined using Mann–Whitney *U* tests for quantitative data and Fisher exact tests for categorical data.

aPTT, activated partial thromboplastin time; ALP, alkaline phosphatase; ALT, alanine aminotransferase; ANC, absolute neutrophil count; AST, aspartate aminotransferase; CRP, C-reactive protein; FUO, fever of unknown origin; γGT, gamma-glutamyl transpeptidase; Hb, hemoglobin; HLH, hemophagocytic lymphohistiocytosis; IgG, immunoglobulin G; LDH, lactate dehydrogenase; PB, peripheral blood; PLT, platelet; PT, prothrombin time; T-Bil, total bilirubin; WBC, white blood cell
