# Supplementary material for: Primary bone marrow lymphoma: A hematological emergency in adults with fever of unknown origin
Source: Cancer Med. 2018 Jul 9;7(8):3713–21. doi: 10.1002/cam4.1669 (PMC6089188; doi:10.1002/cam4.1669)
Supplement: Supplementary file 4 [file CAM4-7-3713-s004.docx]

| **Supplementary Table 4. Early parameters of 48 adults with fever of unknown origin who were subsequently diagnosed with or without primary bone marrow lymphoma (Validation cohort)** | | | |
| --- | --- | --- | --- |
| **Characteristics noted within 3 days of admission** | Immunocompetent adults with FUO^b^ | | *P* value^c^ |
|  | PBML (n=4) | Without PBML (n=44) |  |
| Lymphadenopathy, no. (%) | 0 (0) | 15 (34) | 0.294 |
| Age, years | 78 [67-84] | 67 [49-74] | 0.076 |
| Sex, male | 1 (25) | 31 (71) | 0.101 |
| WBC, /µL (4000–11,000^a^) | 2450 [1725-3100] | 6900 [4500-10950] | 0.009 |
| ANC, /µL (2000–5500^a^) | 1006 [660-1692] | 5075 [2900-7426] | 0.001 |
| Hb, g/dL | 9.4 [8.0-10.5] | 9.4 [8.1-11.6] | 0.725 |
| PLT, × 10^3^/µL (150–400^a^) | 67 [40-81] | 142 [80-247] | 0.017 |
| Leukoerythroblastosis on PB smear | 3 (75) | 6 (14) | 0.017 |
| LDH, IU/L (131–250^a^) | 474 [312-910] | 363 [244-558] | 0.500 |
| CRP, mg/dL (<0.5^a^) | 7.10 [1.48-12.77] | 7.43 [1.50-12.25] | 0.843 |
| Ferritin, ng/mL (4–274^a^) | 2210 [1956-9927] | 1114 [501-3312] | 0.179 |
| IgG, mg/dL (751–1560^a^) | 665 [548-810] | 1400 [1013-1725] | 0.006 |
| T-Bil, mg/dL (0.2–1.6^a^) | 1.44 [1.15-15.80] | 0.61 [0.36-0.89] | 0.027 |
| ALT, U/L (0–40^a^) | 34 [24-44] | 24 [17-42] | 0.511 |
| AST, U/L (5–45^a^) | 54 [47-98] | 39 [20-79] | 0.246 |
| ALP, U/L (10–100^a^) | 281 [219-386] | 81 [60-169] | 0.018 |
| γGT, U/L (M: 8–60; F: 4–51^a^) | 172 [56-366] | 56 [33-112] | 0.126 |
| Na, mmol/L (135–147^a^) | 139 [135-140] | 136 [131-138] | 0.201 |
| PT, sec (8.0–12.0^a^) | 10.9 [10.7-11.5] | 11.5 [10.9-12.6] | 0.163 |
| aPTT, sec (23.9–35.5^a^) | 31.0 [26.3-35.1] | 31.7 [29.2-35.3] | 0.712 |
| Fibrinogen, mg/dL (200–400^a^) | 286 [202-439] | 340 [257-431] | 0.674 |
| Triglycerides, mg/dL (20–200^a^) | 228 [208-302] | 159 [117-217] | 0.062 |
| Splenomegaly | 4 (100) | 19 (43) | 0.046 |
| Hepatomegaly | 0 (0) | 2 (5) | 1.000 |

^a^ normal range

^b^ Values are reported as median [interquartile-range] or n (%)

^c^ Determined using Mann–Whitney *U* tests for quantitative data and Fisher exact tests for categorical data.

aPTT, activated partial thromboplastin time; ALP, alkaline phosphatase; ALT, alanine aminotransferase; ANC, absolute neutrophil count; AST, aspartate aminotransferase; CRP, C-reactive protein; FUO, fever of unknown origin; γGT, gamma-glutamyl transpeptidase; Hb, hemoglobin; HLH, hemophagocytic lymphohistiocytosis; IgG, immunoglobulin G; LDH, lactate dehydrogenase; PB, peripheral blood; PLT, platelet; PT, prothrombin time; T-Bil, total bilirubin; WBC, white blood cell
